# Role of dopamine D1 receptor in the modulation of memory consolidation by passive and self-administered heroin and associated conditioned stimuli

**DOI:** 10.1038/s41598-023-39380-3

**Published:** 2023-08-03

**Authors:** Travis Francis, Francesco Leri

**Affiliations:** Department of Psychology and Collaborative Program in Neuroscience, University of Guelph, Guelph, ON N1G 2W1 Canada

**Keywords:** Classical conditioning, Consolidation, Operant learning, Spatial memory

## Abstract

It has been proposed that opiates modulate memory consolidation, but recent work has indicated that this effect may be mediated by how the drug is experienced (i.e., passive injections vs. self-administration). Because the dopamine (DA) D1 receptor is involved in processing of learning signals and attribution of salience to events experienced by an organism, two studies in male Sprague-Dawley rats tested the effect of blocking this receptor on modulation of memory consolidation by passive and self-administered heroin, in addition to conditioned memory modulation by heroin-paired cues. Using the object location memory task, Study 1 employed SCH23390 (0, 0.05, 0.10 mg/kg, SC) to modulate enhancement of memory consolidation induced by post-training injections of heroin (1 mg/kg, SC) as well as by exposure to the environment paired with heroin injections (6 pairings, 1 h each, 1 mg/kg). Study 2 was conducted in rats that could self-administer heroin (0.05 mg/kg/infusion, IV) and tested whether SCH23390 (0 and 0.1 mg/kg, SC) could prevent memory modulation induced by a change in schedule of self-administration (from fixed to variable ratio). It was found that while repeated passive injections of heroin retained their enhancing effect on memory, when self-administered, heroin enhanced consolidation of object location memory only at the beginning of self-administration and after a change in schedule. Importantly, SCH23390 blocked memory modulation by heroin when passively administered and when the drug was self-administered on a novel schedule. SCH23390 also blocked conditioned memory modulation induced by post-training exposure to heroin-paired cues. Taken together, these results suggest that modulation of memory consolidation by unconditioned and conditioned opiate reinforcers involve a D1-dependent mechanism of salience attribution linked to the anticipation of drug effects.

## Introduction

White and Milner^1^ proposed that reinforcing stimuli promote the acquisition and maintenance of new behaviours by enhancing memory consolidation: a time-dependent process of memory stabilization that is central to learning^2^. In support of this theory, there is significant evidence that chemical reinforcers such as morphine/heroin, cocaine, nicotine, as well as environmental stimuli associated with their effects (conditioned stimuli, CS) enhance memory consolidation in various tasks and species^3–8^. These observations are considered critical to addictive behaviours because drugs that enhance the consolidation of actions performed prior to, or during, the experience of drug effects have the potential to increase the probability that these actions will be repeated in the future^9^.

However, it has been recently observed that only passively received, but not self-administered, heroin enhanced consolidation of object location memory^3^. This is a puzzling observation because intravenous (IV) self-administration of drugs in animals is considered the gold standard for assessing their reinforcing efficacy and addictive potential^10–13^. That is, animals (and humans) clearly consolidate the memories of drug-self administration because, under appropriate conditions, they will continue to engage in behaviours that lead to further drug intake.

A possible approach to follow-up these findings and explore the role of mode of administration on opiate-induced memory modulation may lie in considering the functional characteristics of central dopaminergic (DA) systems. In fact, D1 and D2 receptors in the nucleus accumbens (NAc) are involved in memory formation^14–17^ and our laboratory demonstrated that memory enhancement induced by post-training passive injections of cocaine, nicotine, or exposure to their CSs, can be blocked by a D2 antagonist^18^. Interestingly, although many drugs of abuse, including opiates^19^, increase levels of extracellular DA in the NAc of naïve animals regardless of whether the drug is self-administered or passively received^19–21^, changes in phasic DA levels over repeated exposure are linked to how the drug is administered^20,22–26^. Importantly, phasic DA signalling in the brain has a pivotal role in reinforcement^27,28^ and associative learning^23^. Mediating the incentive signal that promotes reward-seeking^29,30^, phasic DA activity encodes surprising, novel and salient experiences^31–34^ and promotes repetition of actions that immediately precede unanticipated biologically relevant events^33,34^. Moreover, exposure to drug-paired cues in the absence of drug also enhances memory consolidation^3,18^ possibly because of their effects on central DA activity^35–39^ and specific actions on phasic DA transmission^40,41^.

Taken together, these findings suggest the hypothesis that the effect of unconditioned and conditioned opiates on memory consolidation may be modulated by a dopamine-dependent mechanism encoding salience of the experience with the drug and/or cue. Hence, two studies were carried out in male Sprague-Dawley rats tested on an object location memory task that, during the period of memory consolidation (i.e., post-sample), were exposed to: injections of heroin (Study 1); or self-administered heroin (Study 2); or a context paired with the effects of heroin in the absence of the drug (Study 1). Because the D1 receptor is considered key to encoding neural and behavioural responses to unexpected and salient outcomes^42–45^, it was predicted that D1 antagonism by SCH23390^46^ would block the ability of passive heroin, and possibly of a heroin CS, to modulate memory consolidation. Moreover, because a change in reinforcement schedule after development of stable self-administration activates memory stabilization^47–49^, it was also predicted that a shift in reinforcement schedule would rescue the action of self-administered heroin on memory modulation, and that this effect should be blocked by D1 antagonism.

## Methods and materials

### Subjects

One-hundred and sixteen male Sprague-Dawley rats (Charles River, QC) weighing between 250 and 300 g at the beginning of each experiment were individually housed in standard rat cages (polycarbonate; 50.5 × 48.5 × 20 cm) with standard environmental enrichment. Upon arrival, rats were given 1 week of acclimatization to the facility and were maintained on a 12-h reverse light/dark schedule (lights off 7:00 AM, on 7:00 PM). All behavioral testing was conducted during the dark period. Rats had access to 25 g per day of standard rat chow and water ad libitum in their home cages. All procedures were approved by the Animal Care Committee at the University of Guelph and were performed in accordance with recommendations provided by the Canadian Council on Animal Care and ARRIVE guidelines.\

### Surgery

Details of the surgical procedures have been described in Francis et al.^3^. Briefly, rats in Study 2 were surgically implanted with intravenous (IV) silastic catheters (Fisher Scientific, Whitby, ON) in the right jugular vein under general anesthesia induced by isoflurane (4% induction, 2% maintenance). The catheter was passed subcutaneously (SC) in the back of the rat where it exited into a connector (a modified 22-gauge cannula), and flushed daily with saline and every second day with 0.10 ml of a saline–heparin solution. Rats were given at least 7 days to recover from surgery before behavioural testing began.

### Apparatus

#### Operant chambers

Details of the operant chambers have been described in Francis et al.^3^. Briefly, 20 Plexiglas operant chambers (Med Associates, Georgia, VT) each contained a house light, and two levers, one retractable (active) and one stationary (inactive). The active lever entered and remained extended during the entire duration of all sessions, and all responses were recorded. In addition, presses on this lever activated a white light that served as a discrete CS that was paired with heroin delivery in Study 2. The inactive lever served to control for non-specific lever presses; responses on this lever had no consequence but were recorded.

#### Object location

Details of the apparatus have been described in Francis et al.^3^. Briefly, this task assesses the ability of rats to discriminate between familiar and novel locations of objects placed in an open field^50^. The apparatus consisted of an open box (70 cm × 70 cm × 60 cm) made of corrugated plastic. The floor was black and two walls opposite from each other were covered with two distinct patterns while the remaining walls were white. Objects used varied in height, size, and texture. An overhead camera was used to record object exploration of rats while in the apparatus.

### Procedures

#### Passive heroin administration

Rats were injected with 1 mg/kg heroin (SC) and immediately confined in operant chambers for 1 h. This was repeated for each of the 6 (Pavlovian) conditioning sessions in Study 1. The operant chamber was used as a contextual CS to enhance methodological consistency with Study 2. The number of conditioning sessions was selected based on previous findings indicating that such CS elicits approach behavior and modulates memory consolidation when experienced post-training^3^, Rizos et al.^51^; Wolter et al.^7^.

#### Heroin self-administration

In Study 2, rats were placed in the same operant chambers as Study 1, attached to the infusion lines, and allowed to self-administer 0.05 mg/kg/inf heroin (IV) on a fixed ratio 1 (FR1) schedule for a total of 11 daily 3 h sessions. Each session began with activation of the house light, entry of the retractable lever, and illumination of the discrete light CS above the active lever for 30 s. If a rat responded on the active lever during this first period, it received a 150 µl infusion of heroin. Similarly, subsequent presses on the active lever led to heroin infusions and simultaneous illumination of the discrete light CS for 5 s. Responses on the inactive lever were without consequences.

For the 12th 3 h session of self-administration, the schedule of reinforcement was changed to a variable ratio 20 (VR20; heroin infusion obtained, on average, after 20 presses on the active lever). This schedule was chosen because a shift from FR1 to VR20 can generate a DA-dependent prediction error and activate memory stabilization processes^47–49^. The range of active lever presses (1–58) required to achieve an average of 20 responses x inf was based on responses made during the 11th self-administration session.

#### Object location memory task

Prior to receiving heroin in Studies 1 (passive administration) and 2 (self-administration), rats were exposed to the object location apparatus for a single, 10 min habituation session and to the operant chambers for a single, 1 h habituation session. In both studies, animals were then tested for object location memory at key stages of the conditioning/self-administration periods. All tests included a sample and a choice phase. During the sample phase, rats were allowed to explore two identical objects positioned in adjacent corners of the apparatus for a total of 180 s, until 25 s of total object exploration was reached, or whichever came first. Immediately following the sample phase, rats were exposed to heroin or the heroin CS (see below), and then returned to their home cages where they remained undisturbed for 72 h. After this retention delay, they were placed back in the open field for the choice phase with one of the sample objects moved to a new location. This retention interval was chosen as a “suboptimal” condition in which drug naïve rats do not typically express object memory^7,8^. On each test, rats were exposed to new, never-before-seen objects, and the locations of moved objects during the choice phase were counterbalanced for each rat and for each test. Object exploration was defined as the nose pointed directly at the object within 2 cm and/or touching the object with the nose. Time spent investigating objects were scored by an experimenter blind to experimental group allocations.

#### Tests of object memory in Study 1

**Figure 1:**
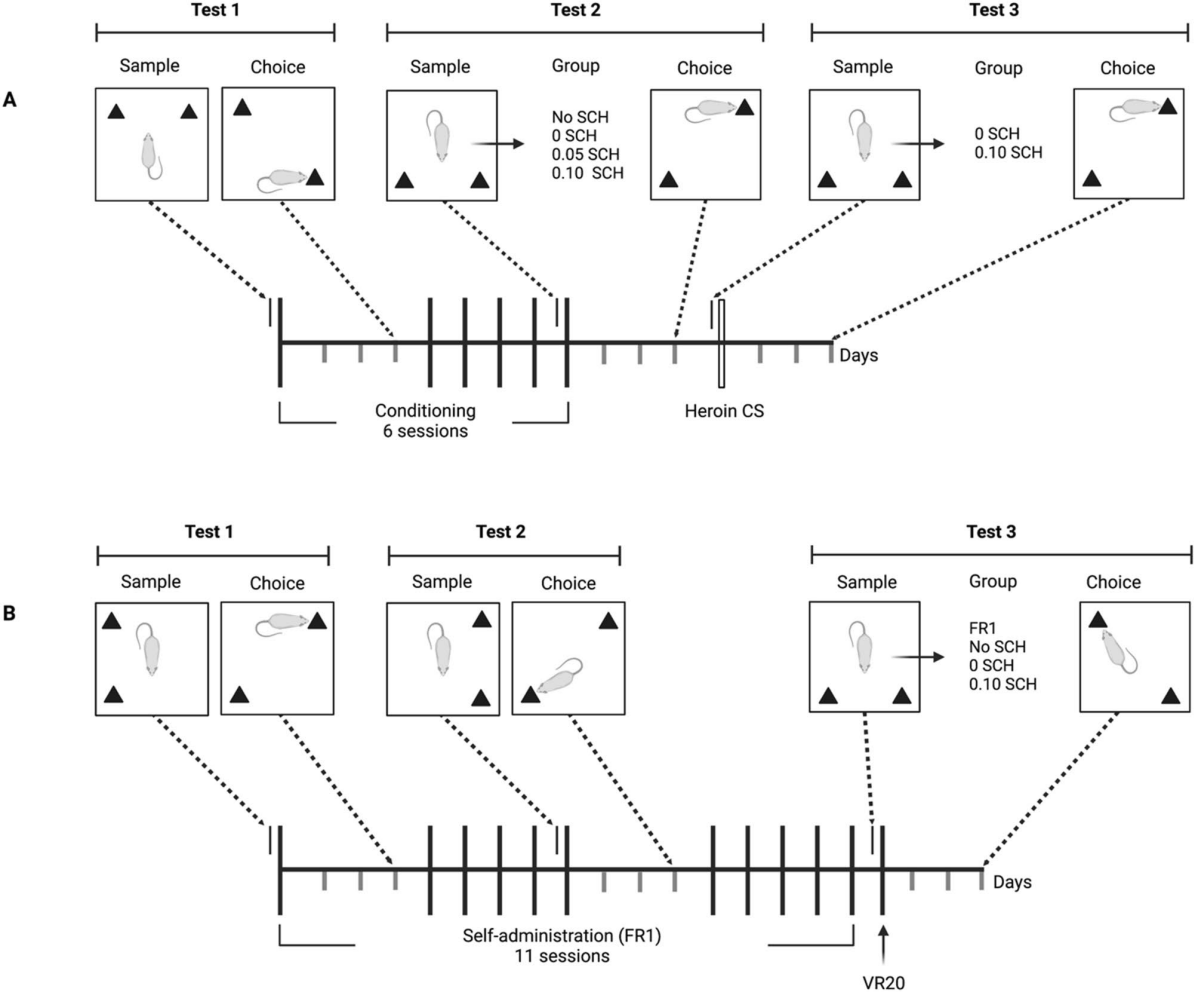
Panel (**A**): experimental design employed in Study 1 showing the relationship between the tests of object location memory and sessions of conditioning/passive heroin injection and exposure to the heroin CS in the absence of heroin. Panel (**B**): experimental design employed in Study 2 showing the relationship between the tests of object location memory and sessions of heroin self-administration.

Panel A of Fig. 1 illustrates how the object location task was employed to explore the effects of SCH23390 (SCH) on memory modulation by passive heroin administration during Pavlovian conditioning and by drug-free exposure to the heroin CS. Test 1 explored whether the 1st conditioning session could modulate object location memory consolidation when no SCH was injected (*n* = 52). The same animals were then re-tested (Test 2) to assess the effects of SCH (0, 0.05, 0.10 mg/kg, *n* = 12 per group) on memory modulation by the 6th session of conditioning. For this test, a subset of rats did not receive SCH (No SCH group; *n* = 16) because they were randomly selected to proceed to Test 3. This final test assessed the effect of SCH (0 and 0.10 mg/kg, *n* = 8 per group) on memory modulation by post-sample exposure (for 1 h) to the heroin CS in the absence of the drug.

Using a separate group of rats (*n* = 8) that was not conditioned, it was verified whether post-training SCH (within-group design, 3 tests, 0, 0.05, 0.10 mg/kg randomized using a Latin Square protocol) could block object location memory by itself using a 24 h retention period; this delay is sufficiently short for drug-naïve rats to display object memory (Winters et al.^52,53^).

#### Test of object memory in Study 2

**Figure 2:**
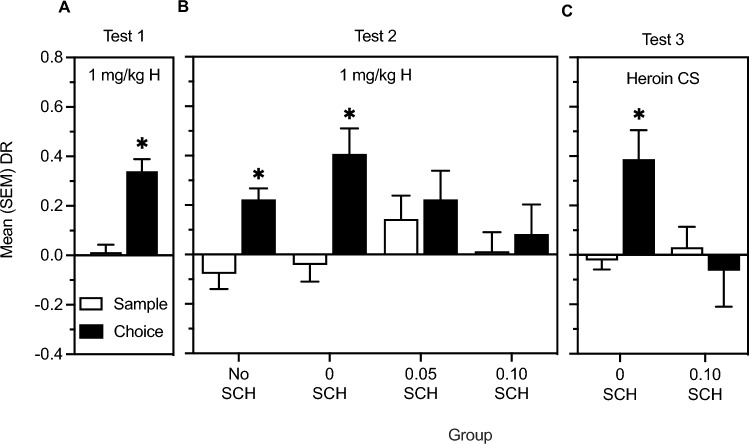
Panel (**A**): mean (SEM) DRs from sample and choice phases of rats (n = 52) administered 1 mg/kg heroin post-sample to test memory modulation by the first conditioning session. Panel (**B**): mean (SEM) DRs from sample and choice phases of rats that received injections of 1 mg/kg heroin but no SCH (No SCH group; *n* = 16), or immediate post-sample injections of either 0, 0.05 or 0.10 mg/kg SCH23390 (*n* = 12 per group) followed by 1 mg/kg injections of heroin. Panel (**C**): mean (SEM) DRs from sample and choice phases of rats that received either 0 or 0.10 mg/kg SCH23390 (*n* = 8 per condition) immediately post-sample followed by exposure to the heroin context CS. The * indicates a significant difference between phases.

Panel B of Fig. 1 illustrates how the object location task was employed to explore whether the modulatory action of self-administered heroin on memory does change over the course of self-administration, and whether the anticipated memory effect caused by a change in reinforcement schedule could be blocked by SCH. Therefore, Tests 1 and 2 assessed, in the same group of rats (*n* = 40), the effect of the 1st and 6th sessions of heroin self-administration on consolidation of object memory, respectively. For the final Test (Test 3), a subset of rats were not injected with SCH (No SCH; *n* = 20) and self-administered heroin on a VR20 schedule. The remaining rats received vehicle or SCH (0 mg/kg, *n* = 10, or 0.10 mg/kg, *n* = 10) prior to the VR20 self-administration session. The 0.10 mg/kg dose of SCH was selected because it was most effective in decreasing choice DRs during Test 2 in Study 1 (Fig. 2B), and a previous locomotion experiment revealed that the impairment in locomotor activity generated by the two doses of SCH (0.05 and 0.10 mg/kg) was similar [SC injection: 0 mg/kg, n = 9; 0.05 mg/kg, n = 8, 0.10 mg/kg, n = 8; total distance moved (cm) in 120 min; 0 mg/kg: Mean = 10,092 SEM = 1,000; 0.05 mg/kg: Mean = 2,830 SEM = 209; 0.10 mg/kg, Mean = 2,511 SEM = 462].

Using a separate group of rats (FR1 group, *n* = 16) similarly trained to self-administer heroin, it was verified whether post-sample exposure to the 12th session of heroin self-administration could modulate object location memory if the FR1 schedule was not changed.

### Drugs

Heroin (Diacetylmorphine hydrochloride, Toronto Research Chemicals, Toronto, ON) and SCH23390 (Sigma-Aldrich, Oakville, ON) were dissolved in 0.9% physiological saline. For Study 1, heroin was injected at 1 mg/kg because this dose can enhance object location memory^3^. For Study 2, heroin was self-administered at 0.05 mg/kg/infusion and a volume of 150 µl/infusion because of previous self-administration studies in our laboratory^54–56^. SCH23390 was injected SC at 0, 0.05 or 0.10 mg/kg because when administered alone, rats display object memory when tested using a 24 h retention delay^57^. Finally, SCH23390 was administered immediately post-training followed by a 15 min delay^57^ before exposure to heroin, or to the heroin CS.

### Data analysis

Analysis of object location memory involved the calculation of a discrimination ratio (DR) in the first minute of the choice phase using the formula: [(time exploring object in novel location − time exploring object in familiar location)/total exploration time]^58^. A score of 0 indicated equal exploration of both objects, while a positive score indicated more time spent investigating the object in the novel location. A sample DR was also calculated using an if/then scenario: (if “the right object is in a novel location” during the choice phase, then [(right object exploration − left object exploration)/total exploration of both objects]. A minimum exploration time was not used in these calculations. For all tests, total object exploration on choice was also analyzed to rule out possible motor effects of drug treatments administered 72 h prior. Because this variable was never significantly different between groups, data are not shown, and statistical analyses not reported.

When possible, the data of study Tests common to all animals were combined and analyzed as a single group. Paired t-tests, one, two and three-factor mixed repeated measures ANOVA followed by post hoc comparisons or multiple simple comparisons, when appropriate, were performed using Statistical Package for the Social Sciences (V28 for Mac, SPSS Inc., IBM) with an alpha = 0.05, unless corrected using the Bonferroni method. In cases where the assumption of sphericity was violated, the Greenhouse–Geisser (GG) corrected *P* value was used.

## Results

### Study 1

Figure 2 represents mean (SEM) sample and choice DRs following immediate post-sample exposure to: (A) conditioning with 1 mg/kg heroin on Test 1; (B) conditioning with 1 mg/kg heroin alone (No SCH), or preceded by 0, 0.05 or 0.10 mg/kg SCH on Test 2; (C) the heroin-paired context preceded by 0 or 0.10 mg/kg SCH on Test 3. The t-test comparing Test 1 sample and choice DRs was significant [*t*(51) = − 5.40, *P* < 0.001]. The t-tests comparing Test 2 sample and choice DRs were significant in the No SCH [*t*(15) = − 5.71, *P* < 0.001] and 0 SCH [*t*(11) = − 2.93, *P* < 0.05] groups. Finally, only the t-test on the 0 SCH group was significant on Test 3 [*t*(7) = − 2.83, *P* = 0.05].

Post-sample administration of 0, 0.05 or 0.10 mg/kg SCH did not change 24 h DRs ([0 mg/kg, sample *Mean* = − 0.03, *SEM* = 0.05; choice *Mean* = 0.43, *SEM* = 0.05; [t(7) = − 5.56, *P* < 0.001]; 0.05 mg/kg, sample *Mean* = − 0.07, *SEM* = 0.05; choice *Mean* = 0.62, *SEM* = 0.05; [t(7) = − 12.1, *P* < 0.001]; 0.10 mg/kg, sample *Mean* = 0.02, *SEM* = 0.04; and choice *Mean* = 0.51, *SEM* = 0.07; [t(7) = − 5.31, P < 0.001]).

### Study 2

At the conclusion of self-administration sessions 1, 6 and 12, catheter patency was confirmed by connecting a piece of tubing to a 1 ml syringe, attaching it to the cannula, and slowly pulling back on the syringe. Catheters were considered patent if blood could be drawn. This verification excluded 2 rats from the No SCH group, and 1 rat from the 0.10 SCH group, leading to a final *n* = 37.

**Figure 3:**
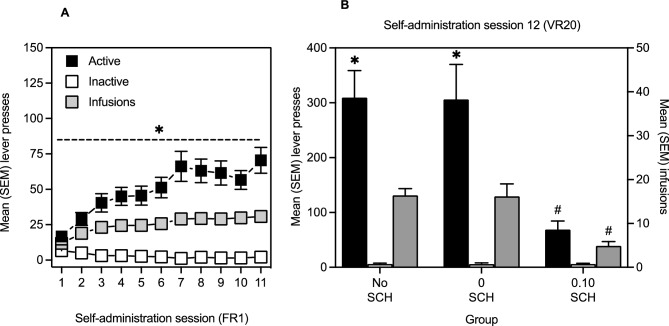
Panel (**A**): mean (SEM) active and inactive lever presses, as well as infusions, during the 11 sessions of heroin self-administration on FR1 (*n* = 37). Panel (**B**): mean (SEM) active and inactive lever presses, as well as infusions, for No SCH (*n* = 18), 0 SCH (*n* = 10), and 0.10 SCH (*n* = 9) groups during the session of self-administration on a VR20 schedule. The * indicates a significant difference between levers. The # indicates a significant difference between groups.

Panel A of Fig. 3 represents mean (SEM) responses on the active and inactive levers during self-administration sessions 1 through 11. The ANOVA revealed a significant Session by Lever interaction [*F*(4.02, 144.8 GG corrected) = 15.5, *P* < 0.001] as well as main effects of Session [*F*(4.00, 144.3 GG corrected) = 10.9, *P* < 0.001] and of Lever [*F*(1, 36) = 60.6, *P* < 0.001]. Multiple comparisons indicated that responses on the active lever were significantly greater than on the inactive lever and that active lever responses significantly increased from Session 1 to Session 11.

Panel A of Fig. 3 also represents infusions delivered during sessions 1 through 11. The t-test comparing number of infusions administered during session 1 versus session 11 was significant [*t*(36) = − 9.00, *P* < 0.001].

Panel B of Fig. 3 represents mean (SEM) responses on the active and inactive levers during the 12th session of heroin self-administration for No SCH, 0 SCH, and 0.10 SCH groups. The ANOVA revealed a significant Lever by Group interaction [*F*(2, 34) = 6.03, *P* < 0.01] as well as main effects of Lever [*F*(1, 34) = 51.4, *P* < 0.001] and of Group [*F*(2, 34) = 5.76, *P* < 0.01]. Multiple comparisons indicated that No SCH and 0 SCH groups responded significantly more on the active than inactive lever, and that both No SCH and 0 SCH groups responded significantly more on the active lever than the 0.10 SCH group.

Panel B of Fig. 3 also represents infusions delivered during session 12 for each group. The one-way ANOVA was significant [*F*(2, 36) = 10.5, *P* < 0.001] and Bonferroni post-hoc comparisons indicated that No SCH and 0 SCH groups self-administered significantly more heroin infusions than the 0.10 SCH group.

**Figure 4:**
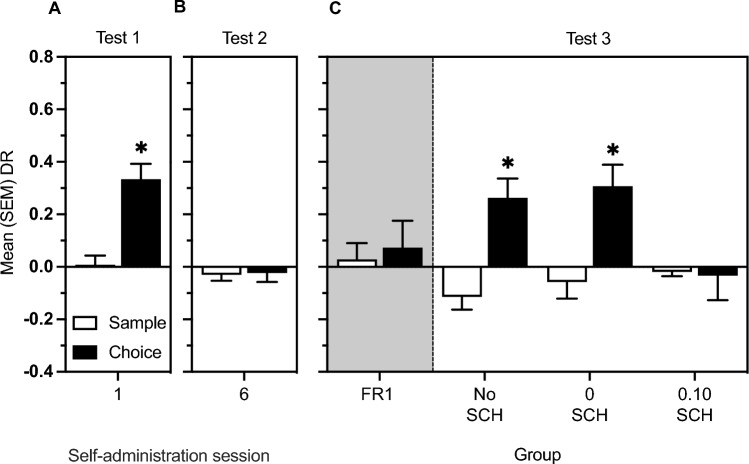
Panels (**A**,**B**): mean (SEM) DRs from sample and choice phases of rats (n = 37) on the test of memory modulation by self-administration sessions 1 and 6, respectively. Panel (**C**): mean (SEM) DRs from sample and choice phases of FR1 only (*n* = 16), No SCH (*n* = 18), 0 SCH (*n* = 10), and 0.10 SCH (*n* = 9) groups on the test of memory modulation by the 12th session of heroin self-administration. The * indicates a significant difference between phases.

Figure 4 represents mean (SEM) sample and choice DRs for Tests 1, 2 and 3, performed in conjunction with the 1st, 6th, and 12th session of heroin self-administration. On Test 1, the t-test comparing sample and choice DRs was significant [*t*(36) = − 4.27, *P* < 0.001]. On Test 2, no significant difference between sample versus choice DRs was found. Finally, on Test 3, t-tests comparing sample and choice DRs were significant only for the No SCH [*t*(17) = − 5.25, *P* < 0.001] and the 0 SCH [*t*(9) = − 3.62, *P* < 0.01] groups.

## Discussion

The primary purpose of the current studies was to test whether the effects of unconditioned and conditioned opiates on memory consolidation involve a dopamine-dependent mechanism encoding salience of the experience with the drug and/or the drug CS. Using male Sprague-Dawley rats and the object location memory task, Study 1 employed the D1 receptor antagonist SCH23390 (0, 0.05, 0.10 mg/kg SC) to modulate enhancement of memory consolidation induced by post-training passive administration of heroin (SC injections of 1 mg/kg) as well as by exposure to the context paired with heroin injections (6 pairings, 1 h each, 1 mg/kg). Study 2 was conducted in animals that could self-administer heroin (0.05 mg/kg/infusion IV), and further explored whether SCH23390 (0 and 0.10 mg/kg SC) could prevent memory modulation induced by a change in schedule of self-administration (from fixed to variable ratio). It was found that while repeated passive administration of heroin retained its enhancing effect on memory, when self-administered, heroin enhanced consolidation of object location only at the beginning of self-administration and after a change in schedule. Importantly, SCH23390 blocked memory modulation by heroin when passively administered and when the drug was self-administered on a novel schedule. SCH23390 also blocked conditioned memory modulation induced by drug-free exposure to the heroin-paired context.

The findings in this report indicate that the effect of heroin on memory consolidation is significantly influenced by the relationship between behaviour and drug delivery. Supporting this conclusion, in Study 1, heroin (1 mg/kg) administered passively as a bolus injection maintained its ability to modulate memory throughout the period of conditioning. However, in rats that were able to self-administer (Study 2), enhanced object location memory was observed after the 1st (Fig. 4A), but not 6th (Fig. 4B), session of self-administration. These findings, which confirm the previous observation that heroin self-administration in well-trained animals does not modulate consolidation of object memory^3^, suggest that heroin’s modulatory effect on memory changes when animals are able to engage in behaviours directly linked to drug delivery. This interpretation is in line with the idea that activation of learning and memory systems occurs as a function of how meaningful, or salient, an event is to the subject^27,29,59–61^. This said, it could be asked whether the larger amount of heroin consumed in later sessions (average intake on 1st session = 0.59 mg/kg; 6th session = 1.3 mg/kg) explains the absence of memory modulation in well-trained animals. This, does not seem to be the case because although post-training administration of opiates can impair discrimination learning and performance in inhibitory avoidance tasks^62,63^, it has been found that when very similar doses of intravenous heroin are delivered passively using a yoked design (i.e., when the behaviour of the animal has no influence on drug delivery), heroin retains its ability to modulate memory^3^.

There are other findings consistent with the idea that responding for heroin can significantly modulate its effect on memory consolidation. In fact, Study 2 established, for the first time, that heroin-induced memory enhancement could be rescued by a shift in reinforcement contingency from FR1 to VR20 on the 12th session of self-administration (Fig. 4C). This again fits the interpretation that the salience of the drug effect was renewed when the contingency between responses and drug delivery was modified. This rescue could not simply be attributed to the longer duration of the self-administration period because a separate group of animals similarly trained to self-administer heroin displayed no evidence of memory facilitation if the FR1 schedule was maintained during the last session of self-administration (Fig. 4C, grey panel, FR1 group). Similarly, although a switch from FR1 to VR20 increased motor output during the session (337% increase in active lever pressing), and increased motor activity has been linked to elevated DA levels^64,65^ that could facilitate memory^66,67^, we have found clear dissociations between number of lever presses and the post-training effects of operant sessions on object location memory consolidation^3^.

The hypothesis that the salience of the drug experience is involved in the ability of heroin to modulate memory consolidation is further supported by the findings with the D1 receptor antagonist SCH23390. In fact, Study 1 determined that immediate post-training injections of 0.05 and 0.10 mg/kg SCH blocked enhancement of object location memory induced by passive heroin injections (Fig. 2B). This finding is consistent with the idea that DA signaling is recruited when learning to discriminate behaviours that lead to salient outcomes^33,34^. As well, 0.10 mg/kg SCH prevented memory modulation induced by a change in schedule of self-administered heroin in Study 2 (Fig. 4C). It is unlikely that this latter effect was due to SCH-induced reduction in lever pressing (Fig. 3C), because heroin intake (0.25 mg/kg) on session 12 was still within the range of heroin doses found to modulate object memory when injected post-sample^3,7^. Moreover, 0.1 mg/kg SCH did not abolish lever pressing but rather prevented the increase in responses engendered by the transition from the FR to the VR schedule [active lever presses—on VR20 on Session 12: *Mean* = 68.9, *SEM* = 15.5; on FR1 on Session 11: *Mean* = 69.0, *SEM* = 16.3].

Interestingly, 0.10 mg/kg SCH also blocked modulation of memory by exposure to the heroin CS (operant chamber) in the absence of heroin (Fig. 2C), and this may be the first evidence of involvement of the D1 receptor in conditioned memory modulation. Here, it is critical to note that the effect of SCH23390 is interpreted as a disruption of memory modulation by heroin and by the heroin CS rather than an effect on memory consolidation per se because post-training administration of 0.05 or 0.10 mg/kg SCH alone did not alter object location memory using a 24 h retention period. Because D1 receptors are known to be involved in encoding a learning signal linked to increased burst firing of DA neurons^43,44,68,69^, and because DA activity is involved in the formation and modulation of memory^15–18^, the current studies suggest that opiate reinforcers enhance memory specifically when their effects are first associated with particular responses, when the contingency between responses and drug delivery is altered, or when exposure to a heroin CS is followed by the absence of heroin delivery.

There are a few limitations to consider when interpreting these results. First, because SCH23390 was administered systemically and D1 receptors are distributed throughout the brain^70^, it is not known which systems of learning and memory were affected. Consequently, it may be valuable to establish whether infusion of SCH23390 into the NAc or ventral tegmental area^65,68,71^ could mimic the effects of systemic administration. Moreover, there is evidence that other neuromodulatory systems, such as noradrenaline, are involved in the effects of opioid agonists on memory consolidation^7,72–74^. As well, because the effect of opioids on DA involves adrenergic inputs^75^, we cannot establish how blockade of D1 receptors may have impacted these other systems. Fourth, because DA levels were not measured in the current study, it is unknown whether changes in DA concentration co-occurred with changes in memory modulation. Optogenetic/DREDD/electrochemical methodologies could provide valuable insights to clarify this important question. Fifth, these studies need replication in female animals, as females acquire opiate self-administration faster, consume more drug, and appear more motivated to consume opioids^76,77^. Lastly, it would be important to repeat these studies using other unconditioned and conditioned drug reinforcers, such as cocaine and nicotine, to determine whether the effects found with heroin can be generalized to other drugs that are self-administered and abused by humans^19^.

## Data Availability

The datasets generated during and/or analysed during the current study are available from the corresponding author upon reasonable request.
